# Designing Implementation Strategies for the Inclusion of Lesbian, Gay, Bisexual, Transgender, Intersex, Queer, and Allied and Key Populations’ Content in Undergraduate Nursing Curricula in KwaZulu-Natal, South Africa: Protocol for a Multimethods Research Project

**DOI:** 10.2196/52250

**Published:** 2024-05-31

**Authors:** Celenkosini Thembelenkosini Nxumalo, Zamasomi Luvuno, Wilbroda Hlolisile Chiya, Silingene Joyce Ngcobo, Deshini Naidoo, Sophia Zamudio-Haas, Orlando Harris

**Affiliations:** 1 Research Development and Postgraduate Support Office of the DVC Research and Innovation University of the Western Cape Cape Town South Africa; 2 Academic Development Unit Faculty of Health Sciences Durban University of Technology Durban South Africa; 3 Centre for Rural Health School of Nursing and Public Health University of KwaZulu-Natal Durban South Africa; 4 Discipline of Nursing School of Nursing and Public Health University of KwaZulu-Natal Durban South Africa; 5 Discipline of Occupational Therapy School of Health Sciences University of KwaZulu-Natal Durban South Africa; 6 Centre for AIDS Prevention Studies University of San Francisco California San Francisco, CA United States; 7 Department of Community Health Systems University of San Francisco California San Francisco, CA United States

**Keywords:** lesbian, gay, bisexual, transgender, intersex, queer, and allied, LGBTQIA+, key populations, nursing curricula, health science education, South Africa, undergraduate, nursing, lesbian, gay, bisexual, and transgender, LGBT, legislation, health care, unmet, barrier, stigma, discrimination, threats, co-design

## Abstract

**Background:**

Lesbian, gay, bisexual, transgender, intersex, queer, and allied (LGBTQIA+) individuals encounter challenges with access and engagement with health services. Studies have reported that LGBTQIA+ individuals experience stigma, discrimination, and health workers’ microaggression when accessing health care. Compelling evidence suggests that the LGBTQIA+ community faces disproportionate rates of HIV infection, mental health disorders, substance abuse, and other noncommunicable diseases. The South African National Strategic Plan for HIV or AIDS, tuberculosis, and sexually transmitted infections, 2023-2028 recognizes the need for providing affirming LGBTQIA+ health care as part of the country’s HIV or AIDS response strategy. However, current anecdotal evidence suggests paucity of LGBTQIA+ and key populations’ health content in the undergraduate health science curricula in South Africa. Moreover, literature reveals a general lack of health worker training regarding the health needs of LGBTQIA+ persons and other key populations such as sex workers, people who inject drugs, and men who have sex with men.

**Objective:**

This study aimed to describe the design of a project that aims at facilitating the inclusion of health content related to the LGBTQIA+ community and other key populations in the undergraduate nursing curricula of KwaZulu-Natal, South Africa.

**Methods:**

A multimethods design encompassing collection of primary and secondary data using multiple qualitative designs and quantitative approaches will be used to generate evidence that will inform the co-design, testing, and scale-up of strategies to facilitate the inclusion of LGBTQIA+ and key populations content in the undergraduate nursing curricula in KwaZulu-Natal, South Africa. Data will be collected using a combination of convenience, purposive, and snowball sampling techniques from LGBTQIA+ persons; academic staff; undergraduate nursing students; and other key populations. Primary data will be collected through individual in-depth interviews, focus groups discussions, and surveys guided by semistructured and structured data collection tools. Data collection and analysis will be an iterative process guided by the respective research design to be adopted. The continuous quality improvement process to be adopted during data gathering and analysis will ensure contextual relevance and sustainability of the resultant co-designed strategies that are to be scaled up as part of the overarching objective of this study.

**Results:**

The proposed study is designed in response to recent contextual empirical evidence highlighting the multiplicity of health challenges experienced by LGBTQIA+ individuals and key populations in relation to health service delivery and access to health care. The potential findings of the study may be appropriate for contributing to the education of nurses as one of the means to ameliorate these problems. Data collection is anticipated to commence in June 2024.

**Conclusions:**

This research has potential implications for nursing education in South Africa and worldwide as it addresses up-to-date problems in the nursing discipline as it pertains to undergraduate students’ preparedness for addressing the unique needs and challenges of the LGBTQIA+ community and other key populations.

**International Registered Report Identifier (IRRID):**

PRR1-10.2196/52250

## Introduction

### Background

South Africa is one of the most progressive African nations when it comes to legislation protecting the human rights of individuals who identify as lesbian, gay, bisexual, transgender, queer, intersex, and allied (LGBTQIA+) [1]. Despite institutionalized rights, evidence suggests that the LGBTQIA+ community continues to encounter stigma, discrimination, and threats to their personal well-being [2-4]. Studies examining the experiences of gender and sexual minorities reveal that the LGBTQIA+ community often faces neglect, harassment, and substandard treatment from health care providers when seeking medical care [5,6]. Moreover, studies report that the LGBTQIA+ community faces several systemic and structural barriers that hinder access to various forms of basic public services including health care, resulting in unmet health needs [7,8]. The obstacles to health care access stem from structural and systemic issues, including insufficient physical resources tailored to the unique needs of this population, stigmatizing attitudes among health care personnel, and a shortage of skilled professionals capable of providing affirming care tailored to their needs [9-11]. Furthermore, literature suggests that the LGBTQIA+ community faces disproportionate rates of HIV or AIDS and tuberculosis infection [12]. Moreover, they are at higher risk for mental health disorders, substance abuse, and long-term sequelae resulting from untreated communicable and noncommunicable disease due to barriers hindering access to health care [10,13,14].

The current reported state of health service delivery as it pertains to the LGBTQIA+ community threatens local and global health policy reforms in terms of universal health coverage and the Sustainable Development Goals. It has been argued that the poor training of health care workers regarding the health needs of this community and the limited provision of appropriate health care may account for challenges faced by the LGBTQIA+ community when accessing health services in South Africa [8,9,11,15]. Although literature on the inclusion of LGBTQIA+ health content in the curricula of health science programs in sub-Saharan Africa, including South Africa, is limited, it suggests that there is little, to no, formal theoretical and clinical training provided to undergraduate health science students [16,17]. It is further suggested that, in instances where content is included, it is often among medical disciplines with no systematic structure, which results in inadequate mastery of essential clinical skills and the knowledge required to enable students to learn how to address the health needs of the LGBTQIA+ community [16,18]. The findings of a curriculum mapping exercise to determine LGBTQIA+ health–related content, pedagogical assessment, and methodology at a selected university in South Africa revealed that the gaps in the inclusion of LGBTQIA+ health content in the curriculum were only evaluated once throughout the duration of the course, with no follow-up, long-term assessment to determine knowledge retention, assess improvement of attitudes, or measure increased levels of comfort. Moreover, the teaching styles adopted did not allow for an awareness and reflection of students’ perceptions in a manner that challenges their biases toward the LGBTQIA+ community [16]. The reported inconsistencies in the curriculum may impact the preparedness of health science graduates for a health system that requires that universal, quality care be provided for all, irrespective of gender identity and sexual orientation. In addition, they may account for the reported challenges experienced by LGBTQIA+ persons in relation to access and quality of health services provided. This subsequently necessitates training interventions to be directed at preservice and in-service health care workers to address the current health service delivery disparities faced by the LGBTQIA+ community [19,20].

The South African health system, which is based on a primary health care approach to service delivery, is nurse led, as nurses constitute the majority workforce [21]. The literature reviewed on the training and inclusion of LGBTQIA+ health needs and care has revealed gaps in relation to how such training is provided. Current evidence suggests poor preparation of South African health care workers to address the health needs of this community [7,8]. This may in turn contribute to unmet health needs and the possibility of poor public health outcomes for South Africa, especially in light of the current incidence and prevalence of communicable and noncommunicable diseases.

The South African National Strategic Plan (NSP) for HIV or AIDS, tuberculosis, and sexually transmitted infections (STIs), 2023-2028 necessitates targeted interventions to be directed toward key populations and LGBTQIA+ persons to achieve HIV or AIDS epidemic control, in line with the Joint United Nations Programme on HIV or AIDS 95-95-95 strategy [22,23]. An essential part of achieving this goal thus consists of facilitating access to quality health care to meet the needs of LGBTQIA+ persons and other key populations. Thus, there is a need to facilitate the inclusion of LGBTQIA+ health content in the undergraduate nursing curricula to ensure that preservice nurses are adequately prepared to address the health needs and challenges of these individuals in line with the principles of universal health coverage and the Sustainable Development Goals [24,25].

The proposed research will thus be conducted to facilitate the inclusion of LGBTQIA+ and key populations’ health content in the undergraduate nursing curricula of higher education institutions in KwaZulu-Natal, South Africa. In this regard, 4 higher education institutions, namely, the University of KwaZulu-Natal, the Durban University of Technology, the University of Zululand, and the KwaZulu-Natal College of Nursing, will form part of the project, as these institutions offer undergraduate nursing programs producing nurses who predominantly serve the KwaZulu-Natal province of South Africa. The proposed study findings have implications for nursing and health science education, and the findings may inform knowledge and skills transfer for the inclusion of LGBTQIA+ and key populations’ health content into the curriculum for all undergraduate nurses in South Africa and could extend to broader health science disciplines at higher education institutions in KwaZulu-Natal and throughout the country. In addition, the study findings have implications for future research in this area and for broader public health issues related to provision of health services for the LGBTQIA+ persons and other key populations. For the purpose of this study, other key populations refer to sex workers, drug users, and men who have sex with men (MSM), as identified in the South African NSP for HIV or AIDS, tuberculosis, and STIs, 2023-2028.

### Aim

This study aims to design implementation strategies for the inclusion of LGBTQIA+ and key populations content in the undergraduate nursing curricula in KwaZulu-Natal, South Africa.

### Objectives

The following sections outline the research objectives and corresponding research questions:

1. To analyze the health needs and practices of service provision for the LGBTQIA+ community and other key populations in KwaZulu-Natal, South Africa
2. To explore the existing practices regarding the inclusion of LGBTQIA+ and key populations health content in the curricula of undergraduate nursing programs in KwaZulu-Natal, South Africa
3. To co-design, test, and evaluate relevant approaches to facilitate the inclusion of LGBTQIA+ and key populations health content in the undergraduate nursing curricula in KwaZulu-Natal, South Africa
4. To spread and scale-up the use of co-designed evidence-based strategies for the inclusion of LGBTQIA+ and key populations health content in the undergraduate nursing curricula in KwaZulu-Natal, South Africa

### Research Questions

In relation to objective 1:

1a. What is the current practice of health service provision for the LGBTQIA+ community and other key populations in KwaZulu-Natal, South Africa?

1b. What are the health needs of the LGBTQIA+ community and other key populations in KwaZulu-Natal, South Africa?

In relation to objective 2:

2a. What is the current available health content on the LGBTQIA+ community and other key populations in the curricula of undergraduate nursing programs in KwaZulu-Natal, South Africa?

2b. What are various academics’ and students’ experiences, understanding, and perceptions regarding the inclusion of health content on the LGBTQIA+ community and other key populations in the undergraduate nursing curricula in KwaZulu-Natal, South Africa?

In relation to objective 3:

3a. What strategies can be codeveloped to facilitate the inclusion of health content on LGBTQIA+ community and other key populations in the undergraduate nursing curricula in KwaZulu-Natal, South Africa?

3b. What are the reported outcomes of testing the codeveloped strategies to facilitate the inclusion of health content on the LGBTQIA+ community and other key populations in the undergraduate nursing curricula in KwaZulu-Natal, South Africa?

In relation to objective 4:

4a. What are the recommended approaches to facilitate spread and scale-up of codeveloped strategies to facilitate the inclusion of health content on the LGBTQIA+ community and other key populations in the undergraduate nursing curricula in KwaZulu-Natal, South Africa?

## Methods

### Study Setting

The study will be conducted within the KwaZulu-Natal province of South Africa, and 4 higher education institutions, namely, the University of KwaZulu-Natal, the Durban University of Technology, the University of Zululand, and the KwaZulu-Natal College of Nursing, will be the sites for data collection. These higher education institutions each presently have an annual size intake of 50 students per year and collectively produce between 120 and 170 new nurse graduates annually. The institutions also have a complement of academic staff with varying qualifications ranging from junior degrees to PhDs, with job functions aligned to respective qualifications as per the national higher education policy and regulations stipulated by the professional body.

### Overarching Design

The study will use a multimethods design, encompassing quantitative and qualitative approaches to collect data from various categories of participants to address the objectives of the research study. The multimethods design allows multiple approaches to data collection, which address the different research objectives and research questions that are related to the broad objectives of the research project. Moreover, the design will also allow concurrent triangulation of data, which will inform the process of co-designing strategies to facilitate the inclusion of health content related to the LGBTQIA+ community and other key populations in the undergraduate nursing curricula in KwaZulu-Natal. The study will thus incorporate multiple research designs to execute the research process. The data that will be generated through the research process will inform the design, testing, and implementation of a relevant strategy to facilitate the inclusion of LGBTQIA+ and key populations content in the undergraduate nursing curricula in KwaZulu-Natal using the process of continuous quality improvement (CQI), as recommended by the Institute of Health Improvement [26]. The collection of data with concurrent data analysis will be an iterative process comprising data triangulation, collation, and collaborative strategy design. The study will thus be conducted in three phases that will entail (1) formative phase, (2) co-design and testing, and (3) spread and scale-up. Table 1 provides an overview of the proposed research methodology and research process to be followed for execution of this study, as informed by the 3 phases of the study. All phases of this study are yet to be carried out and will thus commence in 2024. Before this stage of the project, informal activities were conducted through undocumented sensitivity training sessions. These sessions were initiated in response to contextual anecdotal evidence influenced by the study findings, and they were primarily led by 1 of the principal investigators involved in the study [9,15]. Other work relates to the exploration of nursing students’ knowledge attitudes and perceptions regarding the health needs of sexual and gender minority individuals in KwaZulu-Natal, South Africa [27].

**Table 1:** Summary of methodology and study execution.

Research questions				Design		Population		Sampling and data collection processes
**Phase 1: formative phase**								
	**Objective 1: to analyze the health needs and practices of service provision for the LGBTQIA+^a^ community and other key populations in KZN^b^, South Africa**							
		1a. What is the current practice of health service provision for the LGBTQIA+ community and other key populations in KZN, South Africa?	Scoping review using the framework Arksey and O’Malley [28] Empirical study: qualitative descriptive phenomenological case study		Published and gray literature, including related studies, policies, and guidelines Health care workers, that is, nurses, physicians, clinical associates, and policy makers		Population, intervention, context, and outcomes framework to identify relevant literature Purposive sampling of health care workers and key informants and a combination of focus group discussions and in-depth interviews to collect data	
		1b. What are the health needs of the LGBTQIA+ community and other key populations in KZN, South Africa?	Exploratory qualitative descriptive study		LGBTQIA+ individuals and other key populations (men who have sex with men, sex workers, and people who inject drugs)		Purposive adjusted snowball sampling to identify relevant participants and individual interviews and focus group discussions to collect data	
	**Objective 1.1: to explore the existing practices regarding the inclusion of LGBTQIA+ and key populations health content in the curricula of undergraduate nursing programs in KZN, South Africa**							
		1.1a. What is the current available health content on the LGBTQIA+ community and other key populations in the curricula of undergraduate nursing programs in KZN, South Africa?	Retrospective document analysis		Teaching and learning documents including, but not limited to, relevant teaching, learning and assessment policies, study guides, module descriptors, course contents, and examination papers		A modified curriculum audit tool will be used to assess documents with a review period of 12 months before commencement of data collection	
		1.1b. What are various academics’ and students’ experiences, understanding, and perceptions regarding the inclusion of health content on the LGBTQIA+ community and other key populations in the undergraduate nursing curricula in KZN, South Africa?	A qualitative phenomenographic study		Various categories of academic staff and undergraduate nursing students at all levels of study in the selected higher education institutions		Purposive sapling will be used to collect data using focus group discussions	
**Phase 2: co-design and testing**								
	**Objective 2: to co-design, test, and evaluate relevant approaches to facilitate the inclusion of LGBTQIA+ and key populations health content in the undergraduate nursing curricula in KZN, South Africa**							
		2a. What strategies can be codeveloped to facilitate the inclusion of health content on the LGBTQIA+ community and other key populations in the undergraduate nursing curricula in KZN, South Africa?	Consensus methodology using nominal group technique underpinned by the continuous quality improvement process (Plan-Do-Study-Act)		All participants identified in phase 1 of the study (LGBTQIA+ individuals, academics, nursing students, health care workers, policy makers, and core research team)		Purposive sampling of participants identified in phase 1 of the study and focus group discussions to gather data.	
		2b. What are the reported short-term outcomes of testing the codeveloped strategies to facilitate the inclusion of health content on the LGBTQIA+ community and other key populations in the undergraduate nursing curricula in KZN, South Africa?	Exploratory qualitative descriptive study and quantitative descriptive cross-sectional survey		Academic staff and nursing students in various levels where co-designed interventions will be tested		Purposive sampling will be used to collect data from all participants, and a combination of focus group discussions and individual in-depth interviews will be used to gather qualitative data from participants. Quantitative data will be collected using a tested knowledge, attitude, perception, and awareness survey to assess the short-term outcomes of testing the co-designed strategies for facilitating inclusion of LGBTQIA+ and key populations content in the undergraduate nursing curricula in KZN, South Africa	
**Phase 3: spread and scale-up**								
	**Objective 3: to spread and scale-up the use of co-designed evidence-based strategies for the inclusion of LGBTQIA+ and key populations health content in the undergraduate nursing curricula in KZN, South Africa**							
		3a. What are the recommended approaches to facilitate spread and scale-up of codeveloped strategies to facilitate the inclusion of health content on the LGBTQIA+ community and other key populations in the undergraduate nursing curricula in KZN, South Africa?	Data triangulation method		Data collected from phase 1 and 2 and academic staff in the selected higher education institutions		The core research team will use data informed by phase 1 and phase 2 of the study to develop a relevant approach to scale-up co-designed strategies to facilitate the inclusion of LGBTQIA+ and key populations content in the undergraduate nursing curricula in KZN, South Africa	
		3b. What are the reported outcomes of scaling-up the codeveloped strategies to facilitate the inclusion of health content on the LGBTQIA+ community and other key populations in the undergraduate nursing curricula in KZN, South Africa?	Exploratory qualitative descriptive study and quantitative descriptive cross-sectional survey		Academic staff and nursing students in various levels where co-designed interventions will be tested		Purposive sampling will be used to collect data from all participants, and a combination of focus group discussions and individual in-depth interviews will be used to gather qualitative data from participants. Quantitative data will be collected using a tested knowledge, attitude, perception, and awareness survey to assess the large-scale outcomes of scaling-up the co-designed strategies for facilitating inclusion of LGBTQIA+ and key populations content in the undergraduate nursing curricula in KZN, South Africa	

^a^LGBTQIA+: lesbian, gay, bisexual, transgender, intersex, queer, and allied.

^b^KZN: KwaZulu-Natal.

### Phase 1: Formative Phase

#### Overview

This phase serves to facilitate the situational analysis related to the broad objective of the research project. In phase 1, primary and secondary data will be collected to generate evidence on the health needs and practices of health service provision for the LGBTQIA+ community and other key populations in KwaZulu-Natal, South Africa. This phase will also entail execution of research processes to generate data on the existing LGBTQIA+ and key populations content in the undergraduate nursing curricula in KwaZulu-Natal, South Africa. This phase will thus address the research objectives 1 and 2 together with the related research questions of each objective.

#### Design

During the formative phase (phase 1), primary and secondary data will be collected concurrently, analyzed, and triangulated to inform research processes that will unfold in phase 2 of this study. Multiple research designs will be used to gain comprehensive and holistic data that will contribute to evidence that will inform the design of implementation strategies to facilitate the inclusion of LGBTQIA+ and key populations content in the undergraduate nursing curricula in KwaZulu-Natal, South Africa. Thus, the research designs to be used for phase 1 of this study are as follows: (1) scoping review, (2) exploratory descriptive qualitative study design, (3) descriptive phenomenological case study design, (4) document review through a curriculum mapping exercise, and a (5) qualitative phenomenographic study design. These research designs will collectively inform the execution of the research process that will address objectives 1 and 2 of this proposed study.

To attain information on the health needs and practices of health service provision for the LGBTQIA+ community and other key populations, a systematic scoping review guided by the methodological framework by Arksey and O’Malley [28] will be conducted. An empirical research study will also be conducted using an exploratory qualitative descriptive design to gain a more in-depth understanding of the health needs of LGBTQIA+ individuals and other key populations. A descriptive phenomenological case study will subsequently be conducted to explore various stakeholder experiences regarding service provision for the LGBTQIA+ community and other key populations in KwaZulu-Natal, South Africa. The results of this empirical study along with the findings of the scoping review will be integrated with other data that will be generated to inform the health content on the LGBTQIA+ population and key populations that should be included in the undergraduate nursing curricula in KwaZulu-Natal.

A curriculum mapping exercise will also be conducted to review and determine the current content that is available on the LGBTQIA+ community and other key populations in the undergraduate nursing programs in KwaZulu-Natal. A qualitative phenomenographic study design will be used to explore the qualitative differences in the experiences, understanding, and perceptions of various academics and students regarding the inclusion of content related to the LGBTQIA+ community and other key populations in the undergraduate nursing curricula in KwaZulu-Natal, South Africa.

#### Sampling and Recruitment

As a combination of primary and secondary data will be collected during phase 1 of the project, varying recruitment and data collection processes will be conducted. The secondary data that will be collected in this study will comprise document review of existing research including peer-reviewed and gray literature on the practices of health service provision and health needs of the LGBTQIA+ community and other key populations in KwaZulu-Natal, South Africa. The collection of secondary data will also include a curriculum review exercise that will entail mapping of the existing content that is included on the LGBTQIA+ community and other key populations in the undergraduate nursing curricula at higher education institutions in KwaZulu-Natal, South Africa. In this regard, document review of learning materials; study guides; module descriptors; and relevant institutional teaching, learning, and assessment policies will be performed for all undergraduate nursing programs in the selected institutions identified for data collection.

As part of the empirical study related to the health needs of the LGBTQIA+ population and other key populations in KwaZulu-Natal, South Africa, data will be collected from a purposive sample of individuals from the LGBTQIA+ community and other key populations, namely, MSM, transgender persons, sex workers, and people who inject drugs. To recruit participants for this study, a purposive adjusted snowball sampling technique will be used. Initial recruitment of participants will be facilitated through the mediated access as informed by key informants that are acquainted to the research team. Identification of participants will thus begin purposively, wherein the researchers will identify relevant participants from the LGBTQIA+ community and those that belong to other key populations (namely, sex workers, MSM, etc) from their individual network of acquaintances, which will include peers, family members, community networks, and other spheres of influence. Mediated access to relevant participants will also be facilitated through professional research networks and other related organizations of influence. As the researchers broadly have diverse networks of influence by virtue of differences in geographical location and professional position of influence, a diversity in the participants is expected, which may thus minimize researcher biases.

To explore stakeholders’ experiences regarding service provision for the LGBTQIA+ community and other key populations, purposive sampling of key informants, including health care workers, policy makers, key populations, and the people identifying as LGBTQIA+, will be conducted. Policy makers will be recruited from district and provincial department of health offices. Health care workers will be recruited from primary health facilities within various health districts within the KwaZulu-Natal Department of Health and will include nurse managers, primary health care nurses, enrolled nurses, and nursing assistants. Recruitment of these participants will also be facilitated through relevant line managers and heads of establishment within the various levels of the health system. In this regard, written letters of information will be circulated electronically to provide information of the nature of the study and proposed data collection approaches. Following approval and confirmation by relevant managers, the participants will be contacted through the respective reporting lines, and arrangements will be made to facilitate data collection based on their reported convenience in terms of date, time, and preferred mode of data collection (whether web-based or face to face).

For sampling related to the empirical study of the experiences, understanding, and perceptions of various academics and students regarding the inclusion of content of the LGBTQIA+ community and other key populations, convenience sampling techniques will be used to identify undergraduate nursing students and academic staff in various levels of study. Participants will be approached through academic leaders and relevant level coordinators and lecturers. Recruitment of students will thus take place while students are on campus at the various higher education institutions. Written communication will be providing to the relevant head of department and thereafter disseminated to the various subject lecturers. Arrangements will subsequently be made to collect data from the students based on their availability, as informed by the various heads of department of the undergraduate nursing programs and the relevant lecturers. Students will thus be conveniently approached after attending lecturers, and information about the nature of the study will be provided, and those granting consent will be taken to private offices within the institutions where individual in-depth interviews and focus group discussion sessions will be conducted based on the number of students who consent to participate as convenient sampling processes unfold.

#### Data Collection Processes

All primary data will be collected following the appropriate informed consent processes; data will be collected at settings that are most convenient to participants. A combination of individual in-depth interviews and focus group discussions will be conducted. Web-based and face-to-face methods of data collection will be adopted based on the convenience of participants. The web-based data collection methods will be facilitated through Zoom Video Communications, Inc, Microsoft, WhatsApp Inc, and telephonic interviews, as informed by participant’s convenience. All qualitative data will be collected until saturation is reached, and data analysis will be guided by the qualitative design adopted. The individual in-depth interviews and focus group discussions to be held will be guided by a self-developed interview schedule that is aligned to the respective objectives of this study. Typically, all the semistructured interview guides will comprise 2 sections, namely, participant demographics and guiding interview questions related to the various objectives of the study. All interviews are anticipated to be conducted for approximately 35 to 60 minutes to obtain depth of qualitative data.

The secondary data collection processes will be conducted using the relevant methodological framework and data extraction tools aligned to the objective of the study. For the scoping review component, a self-developed data extraction tool including fields such as author, year of publication, summary of methodology, key findings, recommendation, and limitations will be used to chart the data related to the health needs and practices of service provision of the LGBTQIA+ community and key populations. In this regard, 2 different review processes will be followed culminating in 2 scoping review documents. The curriculum mapping exercise to review the existing content of LGBTQIA+ and key populations will be conducted using a self-developed curriculum review audit tool that assesses the type of content delivered, teaching styles, assessment methods adopted, and learning outcomes associated with the content.

### Phase 2: Co-Design and Testing

During this phase, primary and secondary data generated from phase 1 of this study will be consolidated following application of the relevant data analysis and triangulation processes. The consolidated data will be presented as emerging themes and possible recommendations to inform the design, testing, and implementation of strategies to facilitate the inclusion of LGBTQIA+ and key populations content in the undergraduate nursing curricula in KwaZulu-Natal, South Africa. To operationalize the process of co-designing related to the phase 2 of this study, participants recruited as participants during phase 1 of the study will be contacted to facilitate member checking of triangulated data and emerging results from the data analysis process. The participants will be recruited directly by the researchers of the study using information obtained from phase 1 of the study. Workshop sessions will subsequently be set up to facilitate the co-design of strategies to facilitate the inclusion of LGBTQIA+ and key populations content using data collected from phase 1 through nominal group technique to facilitate consensus. Implementation and testing of co-designed strategies will be facilitated through the CQI process. The Plan-Do-Study-Act cycle embedded within the CQI process will be used to iteratively define, test, and refine the appropriate strategies that may lead to successful implementation of appropriate strategies that will facilitate the inclusion of LGBTQIA+ and key populations content in the undergraduate nursing curricula on the basis of contextual relevance and feasibility. Sustained stakeholder engagement through a series of workshops, engagements, and consensus building sessions will be a key feature of this phase and will serve to ensure co-designed strategies that are relevant for the contextual dynamics of higher education and health service delivery responsiveness within the KwaZulu-Natal context. The precise intervention strategy that will be co-designed for testing is presently not known; however, it is anticipated that the strategy to be developed will be informed by primary and secondary data that will be generated in this study. Moreover, the strategy to be codeveloped will also inform the content to be included; approach to inclusion; assessment strategies; and other approaches related to teaching, learning, and assessment. The anticipated outputs of this phase may thus be teaching content, learning aids, teaching guidelines, and related policy briefs and publications. A crucial aspect of this study’s implementation will involve engaging multiple stakeholders, including members of the LGBTQIA+ community and other key populations. This will ensure that the strategies and content to be included in the curricula are informed by the needs of individuals that the students will eventually have to cater to upon completion of their studies. This may thus facilitate contextualized learning to ensure that nursing students who graduate are able to address the health needs of the LGBTQIA+ community and key populations in KwaZulu-Natal, South Africa.

### Phase 3: Spread and Scale-Up

This phase will be informed by the outcomes of the small-scale testing of strategies that will be co-designed during phase 2 of this study. The outcomes of the testing process will be informed by the CQI process that will be adopted during phase 2. During the spread and scale-up, co-designed strategies will be rolled out at a large scale in the undergraduate nursing programs of the selected higher education institutions in KwaZulu-Natal. In this regard, key stakeholders from the undergraduate nursing programs of the selected institutions will be identified as informed by participants included in the phases 1 and 2 of the study. The relevant gatekeeper processes will be followed, and large-scale adoption of the strategies designed in phase 2 will be scaled up based on the outcomes of the testing process and unique contextual factors affecting teaching and learning at different higher education institutions. The precise format of scale-up remains unknown and will depend on the outcomes of phase 2 of the study, which, in turn, is dependent on the findings of phase 1. However, the anticipated scaling up of the co-designed strategy is expected to inform both the content to be included in nursing curricula and the practices necessary to facilitate their inclusion. The spread and scale-up process will also be monitored through CQI processes that entail routine data collection related to students’ and academic staff’s experiences of the spread and scale-up process. Behavior and attitude change assessment will also be embedded within the scale-up process as part of CQI; this will be facilitated through the administration of quantitative survey tools to students and academic staff on attitudes, perceptions, and awareness regarding key contents related to the LGBTQIA+ community and key populations in KwaZulu-Natal, South Africa. The long-term sustainability of the project and emerging outcomes will be ensured by the development of policy briefs and recommendations for teaching, learning, and assessment that advocate for co-designed content and strategies to be embedded within mainstream curriculum and pedagogical practices based on the contextual dynamics of the specific higher education institutions. In addition, the inclusion of various categories of academic staff from various higher education institutions as part of co-design, testing, and implementation also serves as one of the means of ensuring long-term sustainability.

### Data Analysis

#### Qualitative Data Analysis

The primary qualitative data collected in this study will be analyzed based on the design adopted per objective of the study. All qualitative data collected will be analyzed following verbatim transcription of all interviews that will be conducted by the researchers and contracted research assistants. In line with primary data collection processes, data collected using the exploratory qualitative design will be analyzed inductively through content analysis, as recommended by Cresswell [29]. The analysis of the data will thus include (1) verbatim transcription of all audio-recorded interviews, (2) writing of field notes with subsequent reading for comparison with audio-recorded data to ensure accuracy, (3) repeated reading of transcriptions to attain familiarity with contents and formulate meaning units, (4) selection of informative components of the data, (5) repetition of previous steps to ensure valuable information is not missed, (6) grouping of similar topics together, and (7) formulation of relevant themes and subthemes.

For the descriptive phenomenological case study design, data collected will be analyzed as recommended in the steps of phenomenological data analysis by Colaizzai [30], as cited in the study by Nxumalo et al [31]. The steps to be followed are as follows: (1) repeated reading of transcripts to gain a general sense of its contents; (2) extraction of substantial statements in line with the relevant objective; (3) formulation of meanings from the significant statements derived; (4) sorting of formulated meanings into categories, clusters of themes, and themes; (5) integration of findings into an exhaustive description of the phenomenon being investigated; (6) description of the fundamental structure of the phenomenon; and (7) member checking to validate the descriptive results.

Data collected through the phenomenographic study design will be an iterative process following steps guided by Sjöström and Dahlgren [32]. The first step will entail the familiarization, where transcripts are read several times to become familiar with their contents. The second step will involve a more focused reading to deduce differences and similarities in the transcripts. In the third step, the researchers will identify the key elements in the answers. During the condensation step, only the meaningful and relevant aspects of the transcript will be extrapolated. The fourth step will involve preliminary grouping, with the focus on identifying and classifying similar responses into preliminary groups. These preliminary groups will be reviewed to ascertain whether any other groups convey the same meaning under different headings. This will be followed by a preliminary comparison of categories. Following confirmation, categories were named to highlight their significance. In the final step, the hope is to discover the outcome space based on their internal relationships and the qualitatively unique ways of understanding, conceiving, and experiencing the phenomena. Analysis of secondary data from the curriculum reviews and scoping reviews will be conducted thematically using steps recommended by Braun and Clarke [33]. The secondary data will thus be presented narratively following thematic analysis.

#### Quantitative Data Analysis

The quantitative data collected as part of the CQI process of phase 3 of the study will be analyzed using descriptive statistics and inferential analysis. To facilitate this process, the data collected from Google Forms will be imported into an Excel spreadsheet (Microsoft Corp) in preparation for analysis, which will be conducted using SPSS (IBM Corp). The demographic profile data will be converted into categorical data, and the remaining sections will be converted into statistical data for interpretation. The categorical variables will be analyzed descriptively as counts and percentage frequencies. To determine the association between categorical variables, the chi-square test will be used. When the distribution of the cross-tabulations contains an expected value of <5, the Fischer exact test will be applied.

### Trustworthiness and Rigor

Techniques to ensure dependability, credibility, confirmability, and transferability in this study will include triangulation and member checking. Individual in-depth interviews recorded using an audiotape will ensure credibility and confirmability of the data. Transcribing the data accurately and using appropriate data analysis methods informed by the research design will ensure the dependability of the process. The iterative data collection and concurrent analysis approaches to be adopted will also facilitate credibility and confirmability. Data analysis will be done by experts in qualitative research methods to ensure reliability of findings. Moreover, member checking will also be done to facilitate confirmability. To facilitate member checking, copies of transcriptions with emerging preliminary analyses of the data will be provided electronically to all participants to ascertain whether the transcripts and analyzed data reflect participants’ responses and personal interpretation of the data. Moreover, during the workshop sessions to be held in phase 2 in line with the CQI processes, the analyzed data will be presented for participants to provide input as part of ensuring that the analyzed data presented resonate with the participants’ personal interpretations and intended meaning they wished to convey during the qualitative interviews. All details related to the design and execution of the study are documented to facilitate transferability of the findings that are to emerge. For the quantitative data, the internal consistency of a set of items will be assessed using Cronbach α and the item-rest correlation. Inferential statistical analysis tests will be conducted at a 5% level of significance.

### Ethical Considerations

Ethics approval has been obtained from the Biomedical Research Ethics Committee at the University of KwaZulu-Natal (BREC/00002917/2021). Gatekeeper permission to conduct the study has been obtained from all selected higher education institutions where data will be collected. Written and verbal informed consent will be obtained from all participants before data collection. Ethical principles of autonomy, confidentiality, anonymity, and the right to withdraw will be observed. To ensure autonomy and right to withdraw from the study, informed consent will be obtained from all participants before data collection, and a written information sheet will be provided to all participants clarifying the nature of the study, in addition to the verbal information that will be provided. Informed consent will be obtained verbally from all participants, and they will be given the option to provide voluntary consent and opt out of the study at any time without any form of penalty. Confidentiality and anonymity will be ensured during the data collection processes by conducting individual interviews and focus groups at private venues that are not accessible to the general public; moreover, special precautions will be taken to avoid collecting personal information such as names, addresses, and phone numbers during the recording of interviews. Unique participant codes will be assigned to ensure anonymity during data storage. The data will be kept in a password-protected folder on 1 researcher’s laptop, accessible only to the research team. In the process of disseminating data, no personal information or data revealing participant identity will be included. This applies to all written outputs such as manuscripts and reports that may result from the data collection process.

## Results

The proposed project is informed by the findings of recent contextual studies related to health service provision for the LGBTQIA+ community and key populations in KwaZulu-Natal, South Africa. The findings of these studies have revealed gaps in the training and preparedness of health science graduates to address the unique needs of these individuals in the South African context. Moreover, the South African NSP for HIV or AIDS, tuberculosis and STIs, 2023-2028 necessitates targeted interventions and health care be provided to the LGBTQIA+ community and key populations as part of the HIV or AIDS response strategy. These interventions must be rooted in holistic and comprehensive person-centered care. The aforementioned factors thus necessitate capacity to be created within existing undergraduate health science programs to enable preservice health care providers to be adequately prepared to attend to the unique health needs of the population. As current empirical evidence reveals gaps in the training of health science graduates in this regard, it is necessary that research be conducted to inform co-design of relevant strategies to facilitate the inclusion of LGBTQIA+ and key populations content in the undergraduate nursing curricula in KwaZulu-Natal, South Africa. Data collection is anticipated to commence in June 2024.

In the context of the proposed study, strategies to facilitate the inclusion of health content related to the LGBTQIA+ community and key populations relate to the design and implementation of relevant course content and other relevant teaching, learning, and assessment materials that may support existing curriculum and pedagogical factors. The precise format of strategies and content that is to be developed remains unknown due to the varying contextual dynamics of higher education institutions and diverse health landscape of South Africa. Nonetheless, the codevelopment and implementation process will be underpinned by recommended best practice related to teaching, learning, and assessment practices, particularly in relation to teaching content related to sexual and gender minority groups. In this context, the plan is to systematically integrate strategies and content into the curriculum. This integration will be designed to encourage values clarification, challenging students’ personal biases. Ultimately, this approach aims to address the current issues of stigma and discrimination experienced by LGBTQIA+ persons and other key populations. It is also envisaged that the training methodology and content will foster a culture of empathy and respect among students, thus enabling them to be able to render affirming health care.

## Discussion

### Anticipated Findings

This protocol paper describes the design of an evidence-informed project aimed at facilitating the inclusion of health content related to the LGBTQIA+ community and other key populations in the undergraduate nursing curricula in KwaZulu-Natal, South Africa. A multimethods design encompassing collection of primary and secondary data using multiple qualitative design methods and quantitative approaches will be used to generate data that will be triangulated and integrated to inform the co-design, testing, and scale-up of strategies to facilitate the inclusion of LGBTQIA+ and key populations content in the undergraduate nursing curricula in KwaZulu-Natal, South Africa. Engaging multiple stakeholders and anchoring the process in CQI will ensure the contextual relevance and sustainability of the project outcomes. This includes the spread and scale-up of the co-designed strategies. As informed by the recommendations of previous contextual and international literature, the undoubted severity of the health-related problems of the LGBTQIA+ community and other key populations and the importance of different types of intersectional discrimination against LGBTQIA+ persons, the appropriateness of high-quality education of nurses emerges as a possible means to ameliorate these problems. On the contrary, existing literature alludes to the lack of training of undergraduate nursing students regarding the unique health needs of the LGBTQIA+ population and other key populations with potential consequences of unmet health needs, which may contribute to poor health outcomes for these individuals [34].

The potential for mortality and morbidity associated with untreated communicable and noncommunicable diseases due to challenges in accessing health care among these individuals has significant public health implications. The training of preservice nurses is thus a crucial step toward addressing the reported challenges experienced by the LGBTQIA+ population and other key populations. In this regard, a holistic understanding of the different factors operating in the complex social processes involved in the health care problems of the LGBTQIA+ community may also be important to facilitate a better understanding of these problems and therefore form the basis for appropriate strategies to be designed and implemented. In relation to training of preservice nurses, this information may form the basis upon which content to inform health service provision is designed and implemented. The proposed study uses a multimethods design to elicit data using a multiphase data collection processes through multistakeholder engagement to co-design contextually relevant strategies to facilitate the inclusion of LGBTQIA+ and key populations content in the undergraduate nursing curricula in KwaZulu-Natal.

### Implications

The expected outcomes of this research have implications for nursing education and scholars in South Africa and globally. It tackles current issues within the nursing discipline, particularly regarding undergraduate students’ readiness to address the specific needs and challenges of the LGBTQIA+ community and other key populations. In the South African context, this study has potential implications for informing preservice nurses training on health care provision for the LGBTQIA+ persons and other key populations, as documented in the South African NSP for HIV or AIDS, tuberculosis and STIs, 2023-2028. In addition, the findings of this study have potential implications for all undergraduate nursing programs in the country and may inform knowledge transfer for other health science disciplines that do not have this content formally embedded in their curricula.

### Limitations

The execution of this project will be limited to undergraduate nursing programs in KwaZulu-Natal, South Africa, which may not necessarily be reflective of all undergraduate nursing programs in the country. Moreover, as the study will be limited to undergraduate nursing programs, the findings may not be entirely applicable to other health science programs. Nonetheless, the findings may provide foundational information to guide the co-design of similar strategies in other health science disciplines and undergraduate nursing programs in South Africa.

### Conclusions

This paper describes the design of a multimethods research project aimed at facilitating the inclusion of LGBTQIA+ community and other key populations in the undergraduate nursing curricula in KwaZulu-Natal, South Africa. This project is informed by the findings of previous contextual studies that have revealed challenges faced by the LGBTQIA+ community when accessing health care. Furthermore, evidence indicates that South African health care workers lack training to address LGBTQIA+ health issues both before and during their service. The proposed study findings may thus contribute to addressing this gap and may serve as a means of ameliorating the health challenges faced by LGBTQIA+ persons. The use of the multimethods design and multiple stakeholder engagement including students, academics, members of the LGBTQIA+ community, and other key populations serves as a means of ensuring that contextually relevant strategies are designed. The CQI process to be adopted as part of the co-design and testing phase will serve to ensure relevance of the strategies to be co-designed. The quantitative and qualitative evaluation processes to be adopted will ensure that outcomes of the project are measured in terms of knowledge, attitudes, and perceptions.

## Abbreviations

CQI: continuous quality improvement
LGBTQIA+: lesbian, gay, bisexual, transgender, intersex, queer, and allied
MSM: men who have sex with men
NSP: National Strategic Plan
STI: sexually transmitted infection

## Data Availability

Data sharing is not applicable to this article as no data sets were generated or analyzed during this study.
